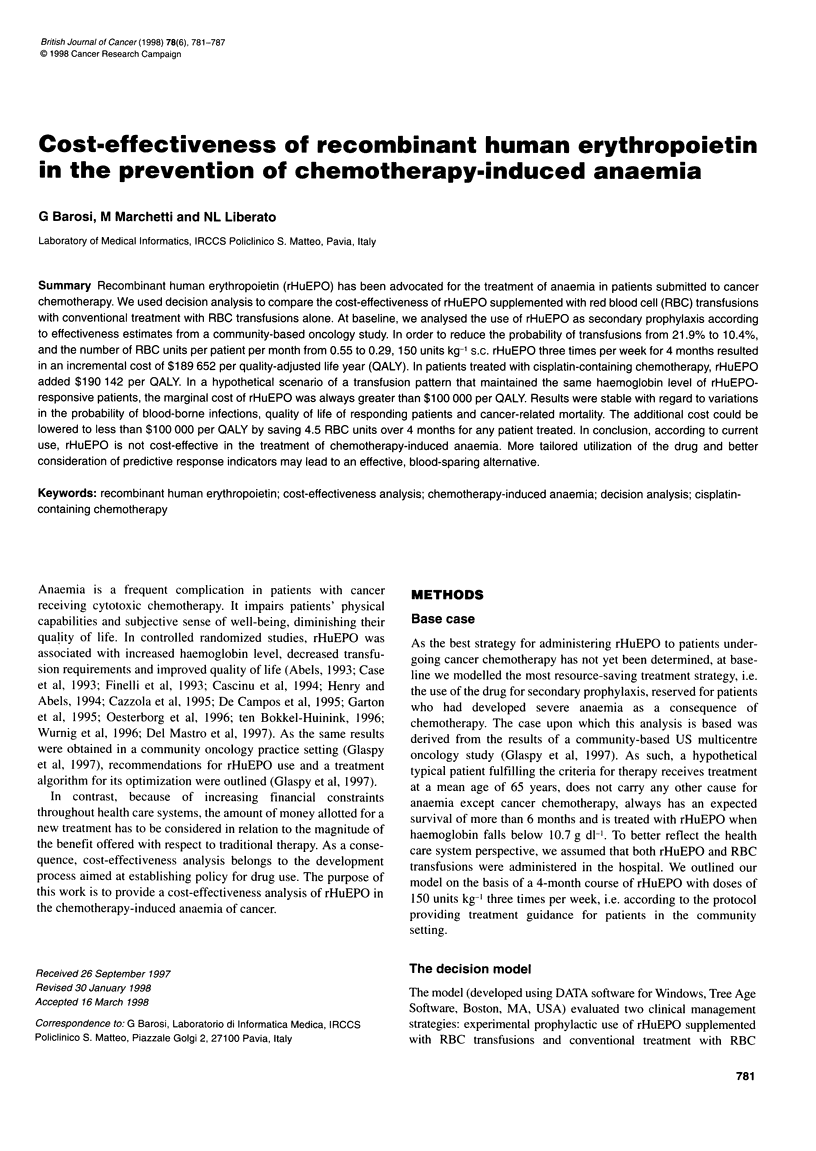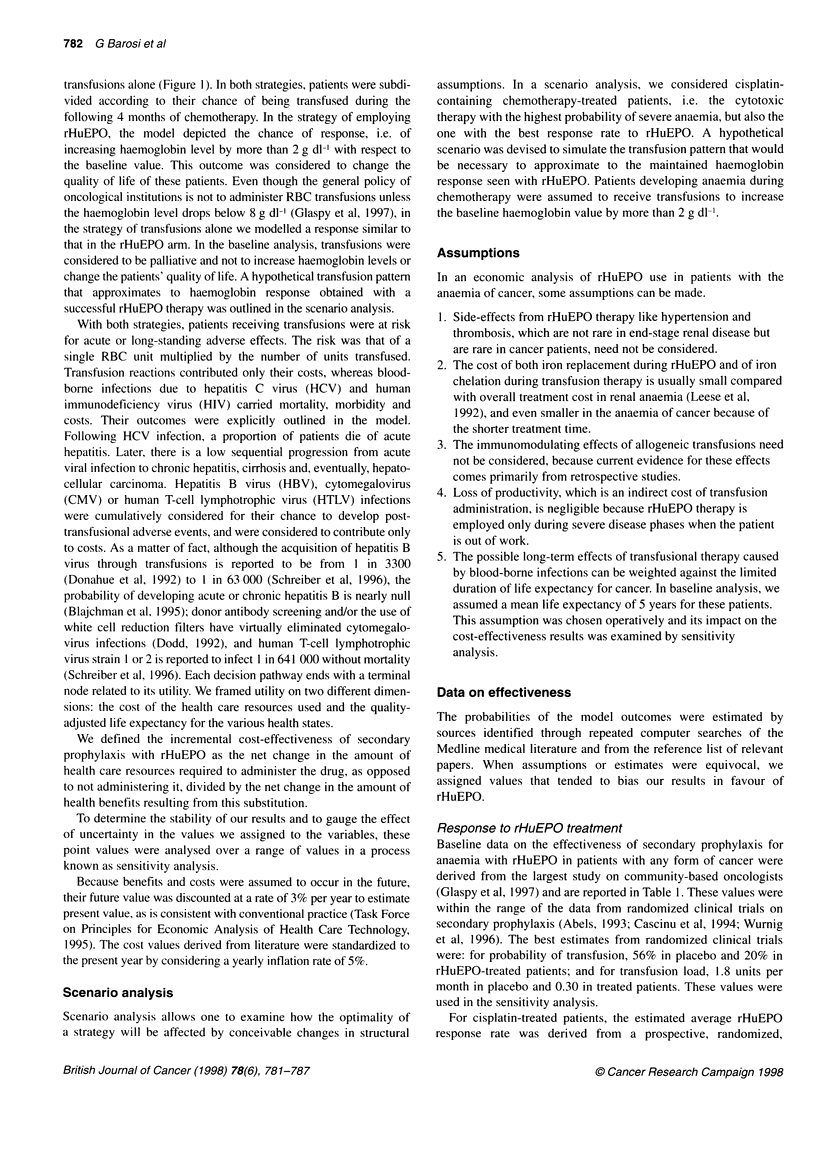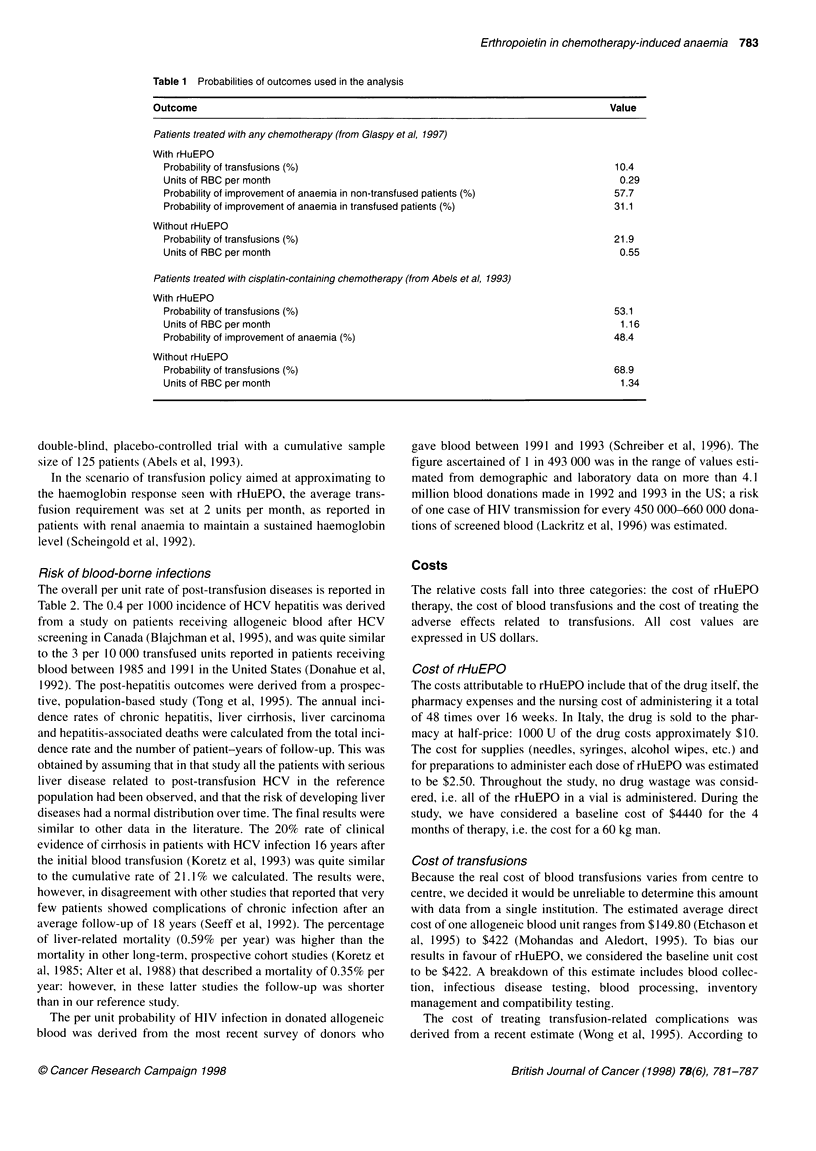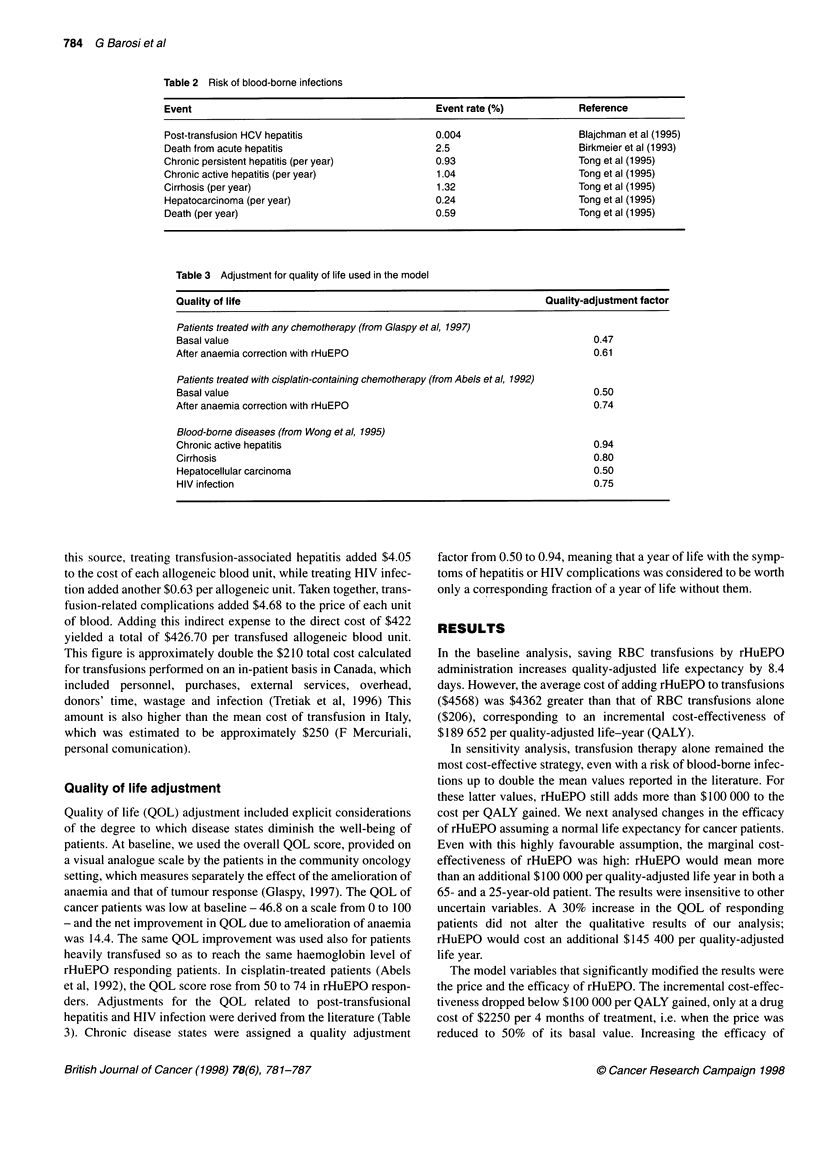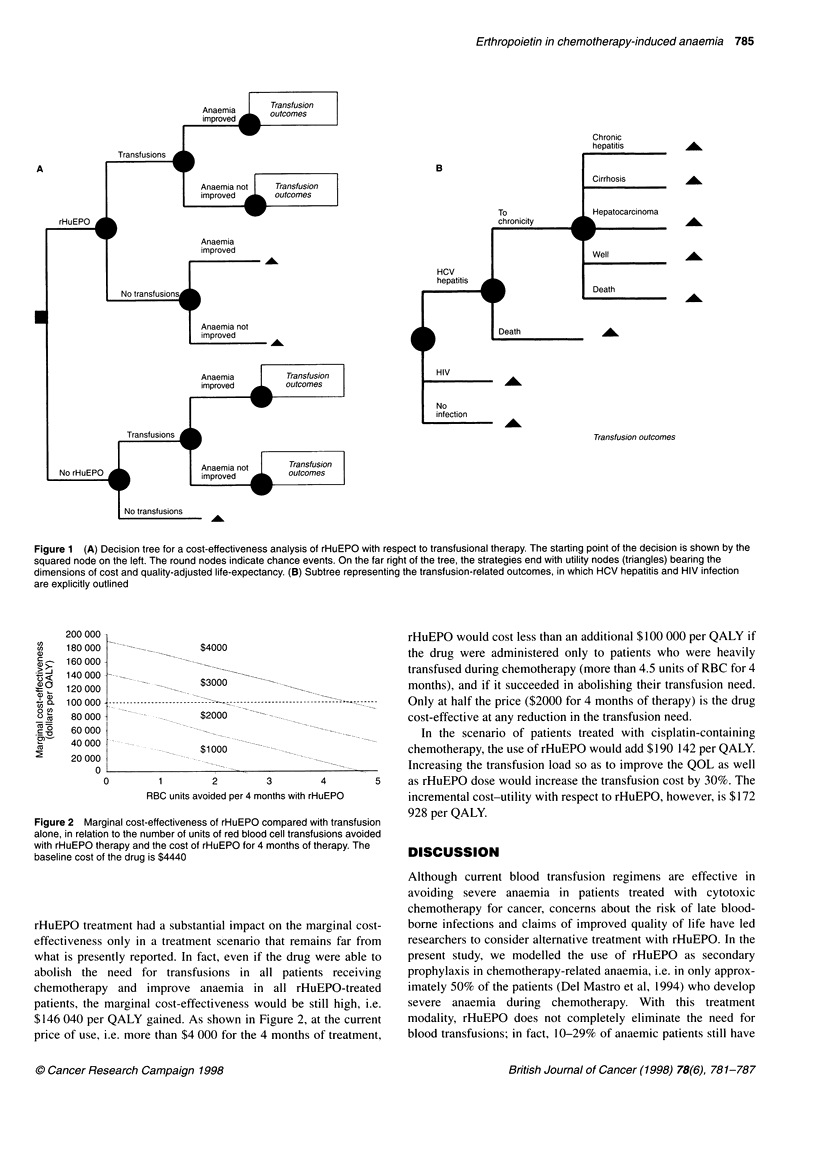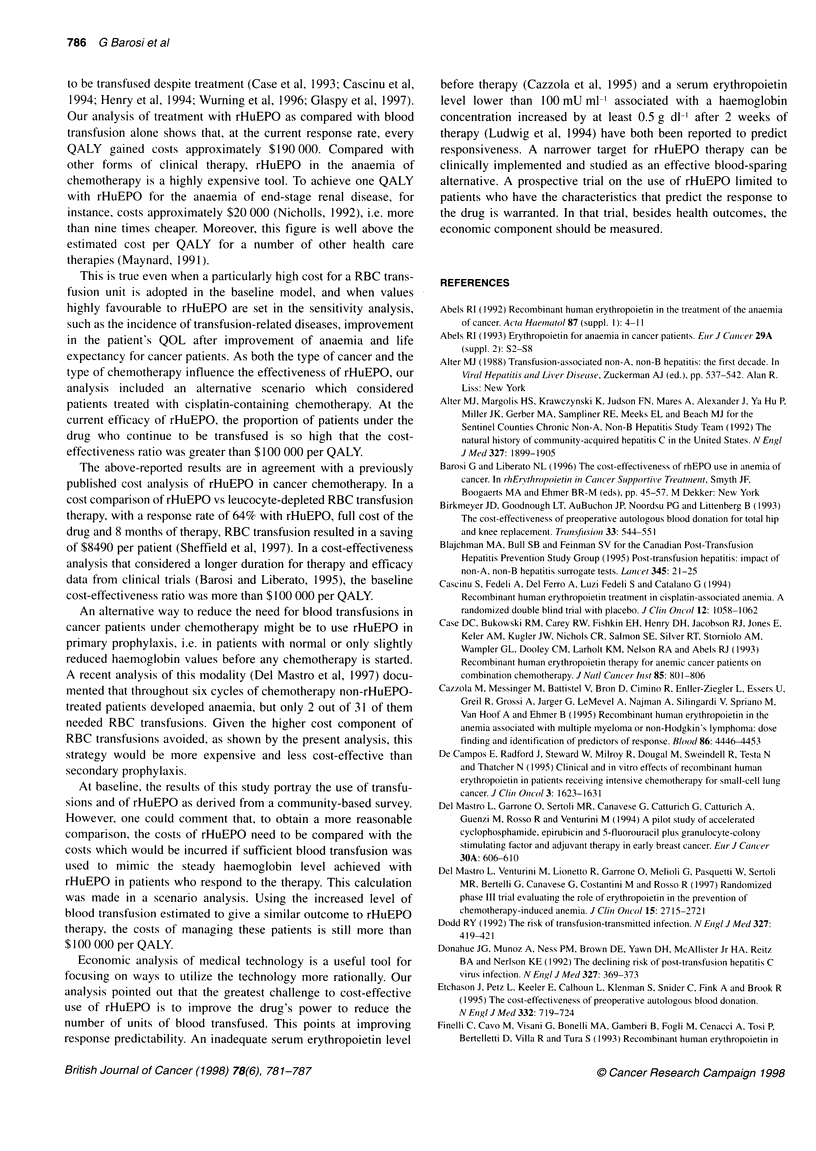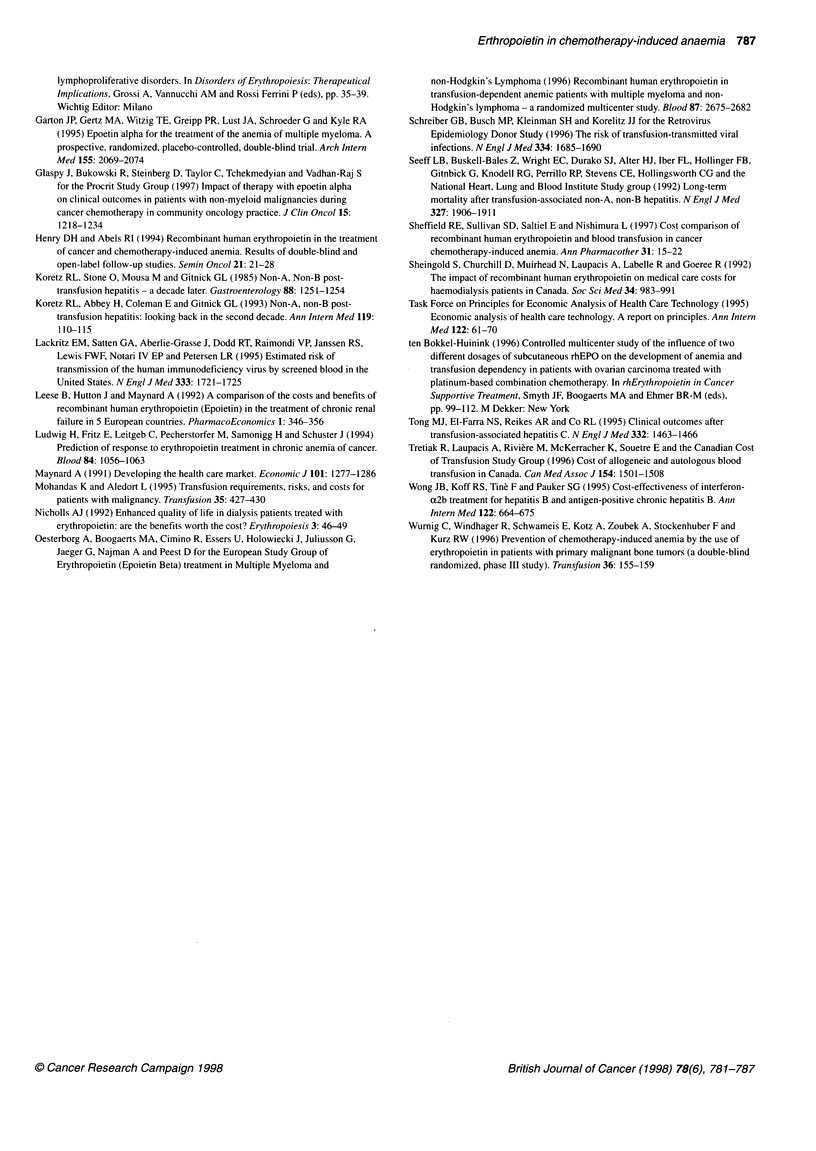# Cost-effectiveness of recombinant human erythropoietin in the prevention of chemotherapy-induced anaemia.

**DOI:** 10.1038/bjc.1998.579

**Published:** 1998-09

**Authors:** G. Barosi, M. Marchetti, N. L. Liberato

**Affiliations:** Laboratory of Medical Informatics, IRCCS Policlinico S. Matteo, Pavia, Italy.

## Abstract

Recombinant human erythropoietin (rHuEPO) has been advocated for the treatment of anaemia in patients submitted to cancer chemotherapy. We used decision analysis to compare the cost-effectiveness of rHuEPO supplemented with red blood cell (RBC) transfusions with conventional treatment with RBC transfusions alone. At baseline, we analysed the use of rHuEPO as secondary prophylaxis according to effectiveness estimates from a community-based oncology study. In order to reduce the probability of transfusions from 21.9% to 10.4%, and the number of RBC units per patient per month from 0.55 to 0.29, 150 units kg(-1) s.c. rHuEPO three times per week for 4 months resulted in an incremental cost of $189,652 per quality-adjusted life year (QALY). In patients treated with cisplatin-containing chemotherapy, rHuEPO added $190,142 per QALY. In a hypothetical scenario of a transfusion pattern that maintained the same haemoglobin level of rHuEPO-responsive patients, the marginal cost of rHuEPO was always greater than $100,000 per QALY. Results were stable with regard to variations in the probability of blood-borne infections, quality of life of responding patients and cancer-related mortality. The additional cost could be lowered to less than $100,000 per QALY by saving 4.5 RBC units over 4 months for any patient treated. In conclusion, according to current use, rHuEPO is not cost-effective in the treatment of chemotherapy-induced anaemia. More tailored utilization of the drug and better consideration of predictive response indicators may lead to an effective, blood-sparing alternative.


British Journal of Cancer (1998) **78**(6), 781–787
© 1998 Cancer Research Campaign

### Cost-effectiveness of recombinant human erythropoietin in the prevention of chemotherapy-induced anaemia

**G Barosi, M Marchetti and NL Liberato**

Laboratory of Medical Informatics, IRCCS Policlinico S. Matteo, Pavia, Italy

**Summary** Recombinant human erythropoietin (rHuEPO) has been advocated for the treatment of anaemia in patients submitted to cancer chemotherapy. We used decision analysis to compare the cost-effectiveness of rHuEPO supplemented with red blood cell (RBC) transfusions with conventional treatment with RBC transfusions alone. At baseline, we analysed the use of rHuEPO as secondary prophylaxis according to effectiveness estimates from a community-based oncology study. In order to reduce the probability of transfusions from 21.9% to 10.4%, and the number of RBC units per patient per month from 0.55 to 0.29, 150 units $kg^{-1}$ s.c. rHuEPO three times per week for 4 months resulted in an incremental cost of $189 652 per quality-adjusted life year (QALY). In patients treated with cisplatin-containing chemotherapy, rHuEPO added $190 142 per QALY. In a hypothetical scenario of a transfusion pattern that maintained the same haemoglobin level of rHuEPO-responsive patients, the marginal cost of rHuEPO was always greater than $100 000 per QALY. Results were stable with regard to variations in the probability of blood-borne infections, quality of life of responding patients and cancer-related mortality. The additional cost could be lowered to less than $100 000 per QALY by saving 4.5 RBC units over 4 months for any patient treated. In conclusion, according to current use, rHuEPO is not cost-effective in the treatment of chemotherapy-induced anaemia. More tailored utilization of the drug and better consideration of predictive response indicators may lead to an effective, blood-sparing alternative.

**Keywords:** recombinant human erythropoietin; cost-effectiveness analysis; chemotherapy-induced anaemia; decision analysis; cisplatin-containing chemotherapy

Anaemia is a frequent complication in patients with cancer receiving cytotoxic chemotherapy. It impairs patients' physical capabilities and subjective sense of well-being, diminishing their quality of life. In controlled randomized studies, rHuEPO was associated with increased haemoglobin level, decreased transfusion requirements and improved quality of life (Abels, 1993; Case et al, 1993; Finelli et al, 1993; Cascinu et al, 1994; Henry and Abels, 1994; Cazzola et al, 1995; De Campos et al, 1995; Garton et al, 1995; Oesterborg et al, 1996; ten Bokkel-Huinink, 1996; Wurnig et al, 1996; Del Mastro et al, 1997). As the same results were obtained in a community oncology practice setting (Glaspy et al, 1997), recommendations for rHuEPO use and a treatment algorithm for its optimization were outlined (Glaspy et al, 1997).

In contrast, because of increasing financial constraints throughout health care systems, the amount of money allotted for a new treatment has to be considered in relation to the magnitude of the benefit offered with respect to traditional therapy. As a consequence, cost-effectiveness analysis belongs to the development process aimed at establishing policy for drug use. The purpose of this work is to provide a cost-effectiveness analysis of rHuEPO in the chemotherapy-induced anaemia of cancer.

*Received 26 September 1997*
*Revised 30 January 1998*
*Accepted 16 March 1998*

*Correspondence to:* G Barosi, Laboratorio di Informatica Medica, IRCCS Policlinico S. Matteo, Piazzale Golgi 2, 27100 Pavia, Italy

### METHODS

### Base case

As the best strategy for administering rHuEPO to patients undergoing cancer chemotherapy has not yet been determined, at baseline we modelled the most resource-saving treatment strategy, i.e. the use of the drug for secondary prophylaxis, reserved for patients who had developed severe anaemia as a consequence of chemotherapy. The case upon which this analysis is based was derived from the results of a community-based US multicentre oncology study (Glaspy et al, 1997). As such, a hypothetical typical patient fulfilling the criteria for therapy receives treatment at a mean age of 65 years, does not carry any other cause for anaemia except cancer chemotherapy, always has an expected survival of more than 6 months and is treated with rHuEPO when haemoglobin falls below 10.7 g $dl^{-1}$. To better reflect the health care system perspective, we assumed that both rHuEPO and RBC transfusions were administered in the hospital. We outlined our model on the basis of a 4-month course of rHuEPO with doses of 150 units $kg^{-1}$ three times per week, i.e. according to the protocol providing treatment guidance for patients in the community setting.

### The decision model

The model (developed using DATA software for Windows, Tree Age Software, Boston, MA, USA) evaluated two clinical management strategies: experimental prophylactic use of rHuEPO supplemented with RBC transfusions and conventional treatment with RBC

transfusions alone (Figure 1). In both strategies, patients were subdivided according to their chance of being transfused during the following 4 months of chemotherapy. In the strategy of employing rHuEPO, the model depicted the chance of response, i.e. of increasing haemoglobin level by more than 2 g $dl^{-1}$ with respect to the baseline value. This outcome was considered to change the quality of life of these patients. Even though the general policy of oncological institutions is not to administer RBC transfusions unless the haemoglobin level drops below 8 g $dl^{-1}$ (Glaspy et al, 1997), in the strategy of transfusions alone we modelled a response similar to that in the rHuEPO arm. In the baseline analysis, transfusions were considered to be palliative and not to increase haemoglobin levels or change the patients' quality of life. A hypothetical transfusion pattern that approximates to haemoglobin response obtained with a successful rHuEPO therapy was outlined in the scenario analysis.

With both strategies, patients receiving transfusions were at risk for acute or long-standing adverse effects. The risk was that of a single RBC unit multiplied by the number of units transfused. Transfusion reactions contributed only their costs, whereas blood-borne infections due to hepatitis C virus (HCV) and human immunodeficiency virus (HIV) carried mortality, morbidity and costs. Their outcomes were explicitly outlined in the model. Following HCV infection, a proportion of patients die of acute hepatitis. Later, there is a low sequential progression from acute viral infection to chronic hepatitis, cirrhosis and, eventually, hepatocellular carcinoma. Hepatitis B virus (HBV), cytomegalovirus (CMV) or human T-cell lymphotrophic virus (HTLV) infections were cumulatively considered for their chance to develop post-transfusional adverse events, and were considered to contribute only to costs. As a matter of fact, although the acquisition of hepatitis B virus through transfusions is reported to be from 1 in 3300 (Donahue et al, 1992) to 1 in 63 000 (Schreiber et al, 1996), the probability of developing acute or chronic hepatitis B is nearly null (Blajchman et al, 1995); donor antibody screening and/or the use of white cell reduction filters have virtually eliminated cytomegalovirus infections (Dodd, 1992), and human T-cell lymphotrophic virus strain 1 or 2 is reported to infect 1 in 641 000 without mortality (Schreiber et al, 1996). Each decision pathway ends with a terminal node related to its utility. We framed utility on two different dimensions: the cost of the health care resources used and the quality-adjusted life expectancy for the various health states.

We defined the incremental cost-effectiveness of secondary prophylaxis with rHuEPO as the net change in the amount of health care resources required to administer the drug, as opposed to not administering it, divided by the net change in the amount of health benefits resulting from this substitution.

To determine the stability of our results and to gauge the effect of uncertainty in the values we assigned to the variables, these point values were analysed over a range of values in a process known as sensitivity analysis.

Because benefits and costs were assumed to occur in the future, their future value was discounted at a rate of 3% per year to estimate present value, as is consistent with conventional practice (Task Force on Principles for Economic Analysis of Health Care Technology, 1995). The cost values derived from literature were standardized to the present year by considering a yearly inflation rate of 5%.

### Scenario analysis

Scenario analysis allows one to examine how the optimality of a strategy will be affected by conceivable changes in structural assumptions. In a scenario analysis, we considered cisplatin-containing chemotherapy-treated patients, i.e. the cytotoxic therapy with the highest probability of severe anaemia, but also the one with the best response rate to rHuEPO. A hypothetical scenario was devised to simulate the transfusion pattern that would be necessary to approximate to the maintained haemoglobin response seen with rHuEPO. Patients developing anaemia during chemotherapy were assumed to receive transfusions to increase the baseline haemoglobin value by more than 2 g $dl^{-1}$.

### Assumptions

In an economic analysis of rHuEPO use in patients with the anaemia of cancer, some assumptions can be made.

1. Side-effects from rHuEPO therapy like hypertension and thrombosis, which are not rare in end-stage renal disease but are rare in cancer patients, need not be considered.
2. The cost of both iron replacement during rHuEPO and of iron chelation during transfusion therapy is usually small compared with overall treatment cost in renal anaemia (Leese et al, 1992), and even smaller in the anaemia of cancer because of the shorter treatment time.
3. The immunomodulating effects of allogeneic transfusions need not be considered, because current evidence for these effects comes primarily from retrospective studies.
4. Loss of productivity, which is an indirect cost of transfusion administration, is negligible because rHuEPO therapy is employed only during severe disease phases when the patient is out of work.
5. The possible long-term effects of transfusional therapy caused by blood-borne infections can be weighted against the limited duration of life expectancy for cancer. In baseline analysis, we assumed a mean life expectancy of 5 years for these patients. This assumption was chosen operatively and its impact on the cost-effectiveness results was examined by sensitivity analysis.

### Data on effectiveness

The probabilities of the model outcomes were estimated by sources identified through repeated computer searches of the Medline medical literature and from the reference list of relevant papers. When assumptions or estimates were equivocal, we assigned values that tended to bias our results in favour of rHuEPO.

### *Response to rHuEPO treatment*

Baseline data on the effectiveness of secondary prophylaxis for anaemia with rHuEPO in patients with any form of cancer were derived from the largest study on community-based oncologists (Glaspy et al, 1997) and are reported in Table 1. These values were within the range of the data from randomized clinical trials on secondary prophylaxis (Abels, 1993; Cascinu et al, 1994; Wurnig et al, 1996). The best estimates from randomized clinical trials were: for probability of transfusion, 56% in placebo and 20% in rHuEPO-treated patients; and for transfusion load, 1.8 units per month in placebo and 0.30 in treated patients. These values were used in the sensitivity analysis.

For cisplatin-treated patients, the estimated average rHuEPO response rate was derived from a prospective, randomized,

© Cancer Research Campaign 1998

**Table 1** Probabilities of outcomes used in the analysis

| Outcome | Value |
|---|---|
| *Patients treated with any chemotherapy (from Glaspy et al, 1997)* | |
| With rHuEPO | |
| Probability of transfusions (%) | 10.4 |
| Units of RBC per month | 0.29 |
| Probability of improvement of anaemia in non-transfused patients (%) | 57.7 |
| Probability of improvement of anaemia in transfused patients (%) | 31.1 |
| Without rHuEPO | |
| Probability of transfusions (%) | 21.9 |
| Units of RBC per month | 0.55 |
| *Patients treated with cisplatin-containing chemotherapy (from Abels et al, 1993)* | |
| With rHuEPO | |
| Probability of transfusions (%) | 53.1 |
| Units of RBC per month | 1.16 |
| Probability of improvement of anaemia (%) | 48.4 |
| Without rHuEPO | |
| Probability of transfusions (%) | 68.9 |
| Units of RBC per month | 1.34 |

double-blind, placebo-controlled trial with a cumulative sample size of 125 patients (Abels et al, 1993).

In the scenario of transfusion policy aimed at approximating to the haemoglobin response seen with rHuEPO, the average transfusion requirement was set at 2 units per month, as reported in patients with renal anaemia to maintain a sustained haemoglobin level (Scheingold et al, 1992).

### *Risk of blood-borne infections*

The overall per unit rate of post-transfusion diseases is reported in Table 2. The 0.4 per 1000 incidence of HCV hepatitis was derived from a study on patients receiving allogeneic blood after HCV screening in Canada (Blajchman et al, 1995), and was quite similar to the 3 per 10 000 transfused units reported in patients receiving blood between 1985 and 1991 in the United States (Donahue et al, 1992). The post-hepatitis outcomes were derived from a prospective, population-based study (Tong et al, 1995). The annual incidence rates of chronic hepatitis, liver cirrhosis, liver carcinoma and hepatitis-associated deaths were calculated from the total incidence rate and the number of patient–years of follow-up. This was obtained by assuming that in that study all the patients with serious liver disease related to post-transfusion HCV in the reference population had been observed, and that the risk of developing liver diseases had a normal distribution over time. The final results were similar to other data in the literature. The 20% rate of clinical evidence of cirrhosis in patients with HCV infection 16 years after the initial blood transfusion (Koretz et al, 1993) was quite similar to the cumulative rate of 21.1% we calculated. The results were, however, in disagreement with other studies that reported that very few patients showed complications of chronic infection after an average follow-up of 18 years (Seeff et al, 1992). The percentage of liver-related mortality (0.59% per year) was higher than the mortality in other long-term, prospective cohort studies (Koretz et al, 1985; Alter et al, 1988) that described a mortality of 0.35% per year: however, in these latter studies the follow-up was shorter than in our reference study.

The per unit probability of HIV infection in donated allogeneic blood was derived from the most recent survey of donors who gave blood between 1991 and 1993 (Schreiber et al, 1996). The figure ascertained of 1 in 493 000 was in the range of values estimated from demographic and laboratory data on more than 4.1 million blood donations made in 1992 and 1993 in the US; a risk of one case of HIV transmission for every 450 000–660 000 donations of screened blood (Lackritz et al, 1996) was estimated.

### Costs

The relative costs fall into three categories: the cost of rHuEPO therapy, the cost of blood transfusions and the cost of treating the adverse effects related to transfusions. All cost values are expressed in US dollars.

### *Cost of rHuEPO*

The costs attributable to rHuEPO include that of the drug itself, the pharmacy expenses and the nursing cost of administering it a total of 48 times over 16 weeks. In Italy, the drug is sold to the pharmacy at half-price: 1000 U of the drug costs approximately $10. The cost for supplies (needles, syringes, alcohol wipes, etc.) and for preparations to administer each dose of rHuEPO was estimated to be $2.50. Throughout the study, no drug wastage was considered, i.e. all of the rHuEPO in a vial is administered. During the study, we have considered a baseline cost of $4440 for the 4 months of therapy, i.e. the cost for a 60 kg man.

### *Cost of transfusions*

Because the real cost of blood transfusions varies from centre to centre, we decided it would be unreliable to determine this amount with data from a single institution. The estimated average direct cost of one allogeneic blood unit ranges from $149.80 (Etchason et al, 1995) to $422 (Mohandas and Aledort, 1995). To bias our results in favour of rHuEPO, we considered the baseline unit cost to be $422. A breakdown of this estimate includes blood collection, infectious disease testing, blood processing, inventory management and compatibility testing.

The cost of treating transfusion-related complications was derived from a recent estimate (Wong et al, 1995). According to

© Cancer Research Campaign 1998

Table 2 Risk of blood-borne infections

| Event | Event rate (%) | Reference |
|---|---|---|
| Post-transfusion HCV hepatitis | 0.004 | Blajchman et al (1995) |
| Death from acute hepatitis | 2.5 | Birkmeier et al (1993) |
| Chronic persistent hepatitis (per year) | 0.93 | Tong et al (1995) |
| Chronic active hepatitis (per year) | 1.04 | Tong et al (1995) |
| Cirrhosis (per year) | 1.32 | Tong et al (1995) |
| Hepatocarcinoma (per year) | 0.24 | Tong et al (1995) |
| Death (per year) | 0.59 | Tong et al (1995) |

Table 3 Adjustment for quality of life used in the model

| Quality of life | Quality-adjustment factor |
|---|---|
| *Patients treated with any chemotherapy (from Glaspy et al, 1997)* | |
| Basal value | 0.47 |
| After anaemia correction with rHuEPO | 0.61 |
| *Patients treated with cisplatin-containing chemotherapy (from Abels et al, 1992)* | |
| Basal value | 0.50 |
| After anaemia correction with rHuEPO | 0.74 |
| *Blood-borne diseases (from Wong et al, 1995)* | |
| Chronic active hepatitis | 0.94 |
| Cirrhosis | 0.80 |
| Hepatocellular carcinoma | 0.50 |
| HIV infection | 0.75 |

this source, treating transfusion-associated hepatitis added \$4.05 to the cost of each allogeneic blood unit, while treating HIV infection added another \$0.63 per allogeneic unit. Taken together, transfusion-related complications added \$4.68 to the price of each unit of blood. Adding this indirect expense to the direct cost of \$422 yielded a total of \$426.70 per transfused allogeneic blood unit. This figure is approximately double the \$210 total cost calculated for transfusions performed on an in-patient basis in Canada, which included personnel, purchases, external services, overhead, donors' time, wastage and infection (Tretiak et al, 1996) This amount is also higher than the mean cost of transfusion in Italy, which was estimated to be approximately \$250 (F Mercuriali, personal comunication).

### Quality of life adjustment

Quality of life (QOL) adjustment included explicit considerations of the degree to which disease states diminish the well-being of patients. At baseline, we used the overall QOL score, provided on a visual analogue scale by the patients in the community oncology setting, which measures separately the effect of the amelioration of anaemia and that of tumour response (Glaspy, 1997). The QOL of cancer patients was low at baseline – 46.8 on a scale from 0 to 100 – and the net improvement in QOL due to amelioration of anaemia was 14.4. The same QOL improvement was used also for patients heavily transfused so as to reach the same haemoglobin level of rHuEPO responding patients. In cisplatin-treated patients (Abels et al, 1992), the QOL score rose from 50 to 74 in rHuEPO responders. Adjustments for the QOL related to post-transfusional hepatitis and HIV infection were derived from the literature (Table 3). Chronic disease states were assigned a quality adjustment factor from 0.50 to 0.94, meaning that a year of life with the symptoms of hepatitis or HIV complications was considered to be worth only a corresponding fraction of a year of life without them.

### RESULTS

In the baseline analysis, saving RBC transfusions by rHuEPO administration increases quality-adjusted life expectancy by 8.4 days. However, the average cost of adding rHuEPO to transfusions (\$4568) was \$4362 greater than that of RBC transfusions alone (\$206), corresponding to an incremental cost-effectiveness of \$189 652 per quality-adjusted life–year (QALY).

In sensitivity analysis, transfusion therapy alone remained the most cost-effective strategy, even with a risk of blood-borne infections up to double the mean values reported in the literature. For these latter values, rHuEPO still adds more than \$100 000 to the cost per QALY gained. We next analysed changes in the efficacy of rHuEPO assuming a normal life expectancy for cancer patients. Even with this highly favourable assumption, the marginal cost-effectiveness of rHuEPO was high: rHuEPO would mean more than an additional \$100 000 per quality-adjusted life year in both a 65- and a 25-year-old patient. The results were insensitive to other uncertain variables. A 30% increase in the QOL of responding patients did not alter the qualitative results of our analysis; rHuEPO would cost an additional \$145 400 per quality-adjusted life year.

The model variables that significantly modified the results were the price and the efficacy of rHuEPO. The incremental cost-effectiveness dropped below \$100 000 per QALY gained, only at a drug cost of \$2250 per 4 months of treatment, i.e. when the price was reduced to 50% of its basal value. Increasing the efficacy of

© Cancer Research Campaign 1998

**Figure 1** (**A**) Decision tree for a cost-effectiveness analysis of rHuEPO with respect to transfusional therapy. The starting point of the decision is shown by the squared node on the left. The round nodes indicate chance events. On the far right of the tree, the strategies end with utility nodes (triangles) bearing the dimensions of cost and quality-adjusted life-expectancy. (**B**) Subtree representing the transfusion-related outcomes, in which HCV hepatitis and HIV infection are explicitly outlined

**Figure 2** Marginal cost-effectiveness of rHuEPO compared with transfusion alone, in relation to the number of units of red blood cell transfusions avoided with rHuEPO therapy and the cost of rHuEPO for 4 months of therapy. The baseline cost of the drug is $4440

rHuEPO treatment had a substantial impact on the marginal cost-effectiveness only in a treatment scenario that remains far from what is presently reported. In fact, even if the drug were able to abolish the need for transfusions in all patients receiving chemotherapy and improve anaemia in all rHuEPO-treated patients, the marginal cost-effectiveness would be still high, i.e. $146 040 per QALY gained. As shown in Figure 2, at the current price of use, i.e. more than $4 000 for the 4 months of treatment, rHuEPO would cost less than an additional $100 000 per QALY if the drug were administered only to patients who were heavily transfused during chemotherapy (more than 4.5 units of RBC for 4 months), and if it succeeded in abolishing their transfusion need. Only at half the price ($2000 for 4 months of therapy) is the drug cost-effective at any reduction in the transfusion need.

In the scenario of patients treated with cisplatin-containing chemotherapy, the use of rHuEPO would add $190 142 per QALY. Increasing the transfusion load so as to improve the QOL as well as rHuEPO dose would increase the transfusion cost by 30%. The incremental cost–utility with respect to rHuEPO, however, is $172 928 per QALY.

### DISCUSSION

Although current blood transfusion regimens are effective in avoiding severe anaemia in patients treated with cytotoxic chemotherapy for cancer, concerns about the risk of late blood-borne infections and claims of improved quality of life have led researchers to consider alternative treatment with rHuEPO. In the present study, we modelled the use of rHuEPO as secondary prophylaxis in chemotherapy-related anaemia, i.e. in only approximately 50% of the patients (Del Mastro et al, 1994) who develop severe anaemia during chemotherapy. With this treatment modality, rHuEPO does not completely eliminate the need for blood transfusions; in fact, 10–29% of anaemic patients still have

© Cancer Research Campaign 1998

to be transfused despite treatment (Case et al, 1993; Cascinu et al, 1994; Henry et al, 1994; Wurning et al, 1996; Glaspy et al, 1997). Our analysis of treatment with rHuEPO as compared with blood transfusion alone shows that, at the current response rate, every QALY gained costs approximately $190 000. Compared with other forms of clinical therapy, rHuEPO in the anaemia of chemotherapy is a highly expensive tool. To achieve one QALY with rHuEPO for the anaemia of end-stage renal disease, for instance, costs approximately $20 000 (Nicholls, 1992), i.e. more than nine times cheaper. Moreover, this figure is well above the estimated cost per QALY for a number of other health care therapies (Maynard, 1991).

This is true even when a particularly high cost for a RBC transfusion unit is adopted in the baseline model, and when values highly favourable to rHuEPO are set in the sensitivity analysis, such as the incidence of transfusion-related diseases, improvement in the patient's QOL after improvement of anaemia and life expectancy for cancer patients. As both the type of cancer and the type of chemotherapy influence the effectiveness of rHuEPO, our analysis included an alternative scenario which considered patients treated with cisplatin-containing chemotherapy. At the current efficacy of rHuEPO, the proportion of patients under the drug who continue to be transfused is so high that the cost-effectiveness ratio was greater than $100 000 per QALY.

The above-reported results are in agreement with a previously published cost analysis of rHuEPO in cancer chemotherapy. In a cost comparison of rHuEPO vs leucocyte-depleted RBC transfusion therapy, with a response rate of 64% with rHuEPO, full cost of the drug and 8 months of therapy, RBC transfusion resulted in a saving of $8490 per patient (Sheffield et al, 1997). In a cost-effectiveness analysis that considered a longer duration for therapy and efficacy data from clinical trials (Barosi and Liberato, 1995), the baseline cost-effectiveness ratio was more than $100 000 per QALY.

An alternative way to reduce the need for blood transfusions in cancer patients under chemotherapy might be to use rHuEPO in primary prophylaxis, i.e. in patients with normal or only slightly reduced haemoglobin values before any chemotherapy is started. A recent analysis of this modality (Del Mastro et al, 1997) documented that throughout six cycles of chemotherapy non-rHuEPO-treated patients developed anaemia, but only 2 out of 31 of them needed RBC transfusions. Given the higher cost component of RBC transfusions avoided, as shown by the present analysis, this strategy would be more expensive and less cost-effective than secondary prophylaxis.

At baseline, the results of this study portray the use of transfusions and of rHuEPO as derived from a community-based survey. However, one could comment that, to obtain a more reasonable comparison, the costs of rHuEPO need to be compared with the costs which would be incurred if sufficient blood transfusion was used to mimic the steady haemoglobin level achieved with rHuEPO in patients who respond to the therapy. This calculation was made in a scenario analysis. Using the increased level of blood transfusion estimated to give a similar outcome to rHuEPO therapy, the costs of managing these patients is still more than $100 000 per QALY.

Economic analysis of medical technology is a useful tool for focusing on ways to utilize the technology more rationally. Our analysis pointed out that the greatest challenge to cost-effective use of rHuEPO is to improve the drug's power to reduce the number of units of blood transfused. This points at improving response predictability. An inadequate serum erythropoietin level before therapy (Cazzola et al, 1995) and a serum erythropoietin level lower than 100 mU $ml^{-1}$ associated with a haemoglobin concentration increased by at least 0.5 g $dl^{-1}$ after 2 weeks of therapy (Ludwig et al, 1994) have both been reported to predict responsiveness. A narrower target for rHuEPO therapy can be clinically implemented and studied as an effective blood-sparing alternative. A prospective trial on the use of rHuEPO limited to patients who have the characteristics that predict the response to the drug is warranted. In that trial, besides health outcomes, the economic component should be measured.

### REFERENCES

Abels RI (1992) Recombinant human erythropoietin in the treatment of the anaemia of cancer. *Acta Haematol* **87** (suppl. 1): 4–11

Abels RI (1993) Erythropoietin for anaemia in cancer patients. *Eur J Cancer* **29A** (suppl. 2): S2–S8

Alter MJ (1988) Transfusion-associated non-A, non-B hepatitis: the first decade. In *Viral Hepatitis and Liver Disease*, Zuckerman AJ (ed.), pp. 537–542. Alan R. Liss: New York

Alter MJ, Margolis HS, Krawczynski K, Judson FN, Mares A, Alexander J, Ya Hu P, Miller JK, Gerber MA, Sampliner RE, Meeks EL and Beach MJ for the Sentinel Counties Chronic Non-A, Non-B Hepatitis Study Team (1992) The natural history of community-acquired hepatitis C in the United States. *N Engl J Med* **327**: 1899–1905

Barosi G and Liberato NL (1996) The cost-effectiveness of rhEPO use in anemia of cancer. In *rhErythropoietin in Cancer Supportive Treatment*, Smyth JF, Boogaerts MA and Ehmer BR-M (eds), pp. 45–57. M Dekker: New York

Birkmeyer JD, Goodnough LT, AuBuchon JP, Noordsu PG and Littenberg B (1993) The cost-effectiveness of preoperative autologous blood donation for total hip and knee replacement. *Transfusion* **33**: 544–551

Blajchman MA, Bull SB and Feinman SV for the Canadian Post-Transfusion Hepatitis Prevention Study Group (1995) Post-transfusion hepatitis: impact of non-A, non-B hepatitis surrogate tests. *Lancet* **345**: 21–25

Cascinu S, Fedeli A, Del Ferro A, Luzi Fedeli S and Catalano G (1994) Recombinant human erythropoietin treatment in cisplatin-associated anemia. A randomized double blind trial with placebo. *J Clin Oncol* **12**: 1058–1062

Case DC, Bukowski RM, Carey RW, Fishkin EH, Henry DH, Jacobson RJ, Jones E, Keler AM, Kugler JW, Nichols CR, Salmon SE, Silver RT, Storniolo AM, Wampler GL, Dooley CM, Larholt KM, Nelson RA and Abels RJ (1993) Recombinant human erythropoietin therapy for anemic cancer patients on combination chemotherapy. *J Natl Cancer Inst* **85**: 801–806

Cazzola M, Messinger M, Battistel V, Bron D, Cimino R, Enller-Ziegler L, Essers U, Greil R, Grossi A, Jarger G, LeMevel A, Najman A, Silingardi V, Spriano M, Van Hoof A and Ehmer B (1995) Recombinant human erythropoietin in the anemia associated with multiple myeloma or non-Hodgkin's lymphoma: dose finding and identification of predictors of response. *Blood* **86**: 4446–4453

De Campos E, Radford J, Steward W, Milroy R, Dougal M, Sweindell R, Testa N and Thatcher N (1995) Clinical and in vitro effects of recombinant human erythropoietin in patients receiving intensive chemotherapy for small-cell lung cancer. *J Clin Oncol* **3**: 1623–1631

Del Mastro L, Garrone O, Sertoli MR, Canavese G, Catturich G, Catturich A, Guenzi M, Rosso R and Venturini M (1994) A pilot study of accelerated cyclophosphamide, epirubicin and 5-fluorouracil plus granulocyte-colony stimulating factor and adjuvant therapy in early breast cancer. *Eur J Cancer* **30A**: 606–610

Del Mastro L, Venturini M, Lionetto R, Garrone O, Melioli G, Pasquetti W, Sertoli MR, Bertelli G, Canavese G, Costantini M and Rosso R (1997) Randomized phase III trial evaluating the role of erythropoietin in the prevention of chemotherapy-induced anemia. *J Clin Oncol* **15**: 2715–2721

Dodd RY (1992) The risk of transfusion-transmitted infection. *N Engl J Med* **327**: 419–421

Donahue JG, Munoz A, Ness PM, Brown DE, Yawn DH, McAllister Jr HA, Reitz BA and Nerlson KE (1992) The declining risk of post-transfusion hepatitis C virus infection. *N Engl J Med* **327**: 369–373

Etchason J, Petz L, Keeler E, Calhoun L, Klenman S, Snider C, Fink A and Brook R (1995) The cost-effectiveness of preoperative autologous blood donation. *N Engl J Med* **332**: 719–724

Finelli C, Cavo M, Visani G, Bonelli MA, Gamberi B, Fogli M, Cenacci A, Tosi P, Bertelletti D, Villa R and Tura S (1993) Recombinant human erythropoietin in

© Cancer Research Campaign 1998

lymphoproliferative disorders. In *Disorders of Erythropoiesis: Therapeutical Implications*, Grossi A, Vannucchi AM and Rossi Ferrini P (eds), pp. 35–39. Wichtig Editor: Milano

Garton JP, Gertz MA, Witzig TE, Greipp PR, Lust JA, Schroeder G and Kyle RA (1995) Epoetin alpha for the treatment of the anemia of multiple myeloma. A prospective, randomized, placebo-controlled, double-blind trial. *Arch Intern Med* **155**: 2069–2074

Glaspy J, Bukowski R, Steinberg D, Taylor C, Tchekmedyian and Vadhan-Raj S for the Procrit Study Group (1997) Impact of therapy with epoetin alpha on clinical outcomes in patients with non-myeloid malignancies during cancer chemotherapy in community oncology practice. *J Clin Oncol* **15**: 1218–1234

Henry DH and Abels RI (1994) Recombinant human erythropoietin in the treatment of cancer and chemotherapy-induced anemia. Results of double-blind and open-label follow-up studies. *Semin Oncol* **21**: 21–28

Koretz RL, Stone O, Mousa M and Gitnick GL (1985) Non-A, Non-B post-transfusion hepatitis – a decade later. *Gastroenterology* **88**: 1251–1254

Koretz RL, Abbey H, Coleman E and Gitnick GL (1993) Non-A, non-B post-transfusion hepatitis: looking back in the second decade. *Ann Intern Med* **119**: 110–115

Lackritz EM, Satten GA, Aberlie-Grasse J, Dodd RT, Raimondi VP, Janssen RS, Lewis FWF, Notari IV EP and Petersen LR (1995) Estimated risk of transmission of the human immunodeficiency virus by screened blood in the United States. *N Engl J Med* **333**: 1721–1725

Leese B, Hutton J and Maynard A (1992) A comparison of the costs and benefits of recombinant human erythropoietin (Epoietin) in the treatment of chronic renal failure in 5 European countries. *PharmacoEconomics* **1**: 346–356

Ludwig H, Fritz E, Leitgeb C, Pecherstorfer M, Samonigg H and Schuster J (1994) Prediction of response to erythropoietin treatment in chronic anemia of cancer. *Blood* **84**: 1056–1063

Maynard A (1991) Developing the health care market. *Economic J* **101**: 1277–1286

Mohandas K and Aledort L (1995) Transfusion requirements, risks, and costs for patients with malignancy. *Transfusion* **35**: 427–430

Nicholls AJ (1992) Enhanced quality of life in dialysis patients treated with erythropoietin: are the benefits worth the cost? *Erythropoiesis* **3**: 46–49

Oesterborg A, Boogaerts MA, Cimino R, Essers U, Holowiecki J, Juliusson G, Jaeger G, Najman A and Peest D for the European Study Group of Erythropoietin (Epoietin Beta) treatment in Multiple Myeloma and non-Hodgkin's Lymphoma (1996) Recombinant human erythropoietin in transfusion-dependent anemic patients with multiple myeloma and non-Hodgkin's lymphoma – a randomized multicenter study. *Blood* **87**: 2675–2682

Schreiber GB, Busch MP, Kleinman SH and Korelitz JJ for the Retrovirus Epidemiology Donor Study (1996) The risk of transfusion-transmitted viral infections. *N Engl J Med* **334**: 1685–1690

Seeff LB, Buskell-Bales Z, Wright EC, Durako SJ, Alter HJ, Iber FL, Hollinger FB, Gitnbick G, Knodell RG, Perrillo RP, Stevens CE, Hollingsworth CG and the National Heart, Lung and Blood Institute Study group (1992) Long-term mortality after transfusion-associated non-A, non-B hepatitis. *N Engl J Med* **327**: 1906–1911

Sheffield RE, Sullivan SD, Saltiel E and Nishimura L (1997) Cost comparison of recombinant human erythropoietin and blood transfusion in cancer chemotherapy-induced anemia. *Ann Pharmacother* **31**: 15–22

Sheingold S, Churchill D, Muirhead N, Laupacis A, Labelle R and Goeree R (1992) The impact of recombinant human erythropoietin on medical care costs for haemodialysis patients in Canada. *Soc Sci Med* **34**: 983–991

Task Force on Principles for Economic Analysis of Health Care Technology (1995) Economic analysis of health care technology. A report on principles. *Ann Intern Med* **122**: 61–70

ten Bokkel-Huinink (1996) Controlled multicenter study of the influence of two different dosages of subcutaneous rhEPO on the development of anemia and transfusion dependency in patients with ovarian carcinoma treated with platinum-based combination chemotherapy. In *rhErythropoietin in Cancer Supportive Treatment*, Smyth JF, Boogaerts MA and Ehmer BR-M (eds), pp. 99–112. M Dekker: New York

Tong MJ, El-Farra NS, Reikes AR and Co RL (1995) Clinical outcomes after transfusion-associated hepatitis C. *N Engl J Med* **332**: 1463–1466

Tretiak R, Laupacis A, Rivière M, McKerracher K, Souetre E and the Canadian Cost of Transfusion Study Group (1996) Cost of allogeneic and autologous blood transfusion in Canada. *Can Med Assoc J* **154**: 1501–1508

Wong JB, Koff RS, Tinè F and Pauker SG (1995) Cost-effectiveness of interferon-α2b treatment for hepatitis B and antigen-positive chronic hepatitis B. *Ann Intern Med* **122**: 664–675

Wurnig C, Windhager R, Schwameis E, Kotz A, Zoubek A, Stockenhuber F and Kurz RW (1996) Prevention of chemotherapy-induced anemia by the use of erythropoietin in patients with primary malignant bone tumors (a double-blind randomized, phase III study). *Transfusion* **36**: 155–159

© Cancer Research Campaign 1998